# Significance of the Wnt signaling pathway in coronary artery atherosclerosis

**DOI:** 10.3389/fcvm.2024.1360380

**Published:** 2024-03-22

**Authors:** Kashif Khan, Bin Yu, Jean-Claude Tardif, Eric Rhéaume, Hamood Al-Kindi, Sabin Filimon, Cristina Pop, Jacques Genest, Renzo Cecere, Adel Schwertani

**Affiliations:** ^1^ Cardiology and Cardiac Surgery, McGill University Health Center, Montreal, QC, Canada; ^2^ Department of Medicine, Montreal Heart Institute, Montreal, QC, Canada

**Keywords:** human, vascular smooth muscle cells, cholesterol efflux, immunohistochemistry, RT-PCR, cholesterol efflux

## Abstract

**Introduction:**

The progression of coronary atherosclerosis is an active and regulated process. The Wnt signaling pathway is thought to play an active role in the pathogenesis of several cardiovascular diseases; however, a better understanding of this system in atherosclerosis is yet to be unraveled.

**Methods:**

In this study, real-time quantitative reverse transcriptase-polymerase chain reaction (RT-PCR) and Western blotting were used to quantify the expression of Wnt3a, Wnt5a, and Wnt5b in the human coronary plaque, and immunohistochemistry was used to identify sites of local expression. To determine the pathologic significance of increased Wnt, human vascular smooth muscle cells (vSMCs) were treated with Wnt3a, Wnt5a, and Wnt5b recombinant proteins and assessed for changes in cell differentiation and function.

**Results:**

RT-PCR and Western blotting showed a significant increase in the expression of Wnt3a, Wnt5a, Wnt5b, and their receptors in diseased coronary arteries compared with that in non-diseased coronary arteries. Immunohistochemistry revealed an abundant expression of Wnt3a and Wnt5b in diseased coronary arteries, which contrasted with little or no signals in normal coronary arteries. Immunostaining of Wnt3a and Wnt5b was found largely in inflammatory cells and myointimal cells. The treatment of vSMCs with Wnt3a, Wnt5a, and Wnt5b resulted in increased vSMC differentiation, migration, calcification, oxidative stress, and impaired cholesterol handling.

**Conclusions:**

This study demonstrates the upregulation of three important members of canonical and non-canonical Wnt signaling pathways and their receptors in coronary atherosclerosis and shows an important role for these molecules in plaque development through increased cellular remodeling and impaired cholesterol handling.

## Introduction

The progression of coronary atherosclerosis is a highly complex and regulated process ranging from intimal thickening to plaque formation and rupture (1). Years of exposure to several risk factors, including dyslipidemia, hypertension, diabetes, and consumption of a Western diet, can lead to a sustained inflammatory response and endothelial dysfunction (1, 2). Chronic activation of the endothelium primes the vasculature to accumulate extracellular lipids in the vessel walls and promotes the migration of activated vascular smooth muscle cells (vSMCs), called myointimal cells, and inflammatory cells into the intima (3). The sustained intraplaque cell migration, proliferation, and expansion eventually lead to rupture, accounting for approximately 50% of myocardial infarctions (1). Although coronary atherosclerosis is well characterized, its molecular pathogenesis is poorly understood. Unraveling the molecular pathways that contribute to the disease is an important step in discovering novel therapies to aid in its prevention and treatment.

The Wnt signaling pathway is an evolutionarily conserved mechanism involved in embryogenesis and organ repair and is implicated in several disease contexts (4–7). There are currently 19 Wnt glycoproteins that belong to either canonical or non-canonical Wnt signaling pathways. The binding of canonical Wnt ligands to Frizzled receptors and lipoprotein-related peptide 5/6 (LRP5/6) coreceptors on the plasma membrane initiate a cascade of events that promotes the nuclear translocation of the transcription factor β-catenin, resulting in the activation of genes involved in cell proliferation, migration, and differentiation. There are also several non-canonical Wnt signaling cascades; however, the most well-studied are the cell polarity and calcium pathways. The non-canonical cell polarity pathway involves the activation of Rho and Rac GTPases to initiate changes in the cytoskeleton, while the non-canonical calcium pathway involves the activation of genes involved in osteogenic differentiation and calcification through the activation of the transcription factor nuclear factor kappa-light-chain-enhancer of activated B cells (NF-κB).

The relationship between Wnt signaling and coronary artery disease (CAD) was first made evident through the discovery of a mutation in the LRP6 coreceptor, in which hypertensive patients with this mutation were more likely to develop CAD (8). Today, several Wnt ligands are under investigation to better understand their role in endothelial dysfunction, SMC migration, monocyte adhesion and intravasation, and foam cell formation (7, 9–11). The treatment of endothelial cells with Wnt5a induced an NF-kB-dependent inflammatory response (12). Wnt5a was shown to induce lipid accumulation, inflammation, and foam cell formation in macrophages (13, 14). Furthermore, Wnt5a was found to be significantly expressed in arterial lesions and elevated in the plasma of carotid atherosclerosis patients compared with healthy controls (15–17). Since Wnt5a belongs to the non-canonical Wnt signaling pathway, there remains a clear need to investigate not only canonical Wnt signaling in plaque development but also other non-canonical Wnt ligands with different downstream signaling pathways (18). Interestingly, upregulating β-catenin, the effector transcription factor in canonical Wnt signaling, was found to induce vSMC proliferation and migration, a hallmark of atherosclerosis (19). Furthermore, Wnt3a was previously shown to induce the proliferation of rat vSMCs through canonical activation (20). There are also reports of Wnt5b playing an active role in intramembranous ossification through non-canonical activation (21–23).

## Aim

Our selection of Wnt3a, Wnt5a, and Wnt5b was strategic, focusing on their distinct roles in coronary atherosclerosis. Wnt3a was chosen for its well-documented influence on vSMC proliferation via the canonical pathway, critical in plaque formation. Wnt5a, as a key player in non-canonical pathways, has been implicated in inflammation and foam cell formation, processes vital to atherosclerotic progression. Wnt5b, although less studied, is associated with calcification in plaques, mirroring its role in bone formation. Expanding the relevance of these molecules in vSMC pathology is critical to understanding the mechanisms by which vSMCs contribute to atherogenesis.

## Materials and methods

### Tissue collection

Coronary artery tissues were obtained in accordance with the McGill University Health Centre guidelines. This study was approved by the Research Ethics Board of McGill University Health Centre and Montreal Heart Institute. We obtained written informed consent from all participants involved in this study. Healthy and diseased coronary arteries were obtained from heart transplants or autopsies between 2009 and 2012 at the McGill University Health Centre and Montreal Heart Institute. Fifty-eight coronary artery plaques were collected for examination. A detailed clinical history of the tissues can be found in Table 1. Tissues were either snap-frozen in liquid nitrogen or fixed in formalin and embedded in paraffin blocks.

**Table 1:** Clinical characteristics of coronary artery disease patients used in the immunohistochemistry studies.

Clinical parameter	Mean ± SD
Total number of cases (*n*)	58
Age (years)	70 ± 14
Sex (male: female)	36:22
Weight (kg)	79.3 ± 18.0
BMI (kg/m^2^)	27.6 ± 5.9
Hypertension (yes:no)	23:7
High cholesterol (yes:no)	26:3
Diabetes (yes:no)	21:11
Ever smoker (yes:no)	13:8
High cholesterol medication (yes:no)	23:2
Statins (yes:no)	23:3
Total cholesterol (mmol/L)	3.8 ± 1.2
LDL (mmol/L)	2.3 ± 1.0
HDL (mmol/L)	0.9 ± 0.3
Triglycerides (mmol/L)	1.7 ± 0.9
Symptomatic (yes:no)	21:16
Stable (yes:no)	29:14
Hemoglobin (g/L)	112.2 ± 20.5
HbA1c (%)	6.6 ± 1.2
Albumin (g/L)	30.6 ± 7.1
Creatinine (µmol/L)	125.8 ± 84.3
Calcium (mmol/L)	2.1 ± 0.2
Aortic valve area (cm^2^)	2.4 ± 1.1
Jet velocity (cm/s^2^)	184.3 ± 94.7
Pmax (mmHg)	16.8 ± 22.1
Pmean (mmHg)	7.0 ± 5.2
LVEF (%)	50.6 ± 18.9
SBP (mmHg)	136.6 ± 22.4
DBP (mmHg)	75.8 ± 10.9
MAP (mmHg)	96.0 ± 12.7

BMI, body mass index; LDL, low-density lipoprotein; HDL, high-density lipoprotein; HbA1c, hemoglobin A1c; Pmax, max pressure across aortic valve; Pmean, mean pressure across aortic valve; LVEF, left ventricular ejection fraction; SBP, systolic blood pressure; DBP, diastolic blood pressure; MAP, mean arterial pressure.

### Tissue expression of Wnt mRNAs

Total RNAs were extracted from snap-frozen normal arteries (*N* = 10) and coronary plaques (*N* = 14) using the TRIzol (Invitrogen)/RNeasy Mini Kit (QIAGEN) combining protocol as previously described (24). Briefly, the first-strand cDNAs were synthesized using 1 µg of total RNAs with the iScript™ cDNA Synthesis Kit (Bio-Rad). The Real-Time qPCR was done using Advanced qPCR mastermix with SUPERGREEN Hi-ROX (Wisent Bioproducts) on the StepOnePlus™ Real-Time PCR System (Applied Biosystems™). The data were analyzed using PrimePCR Analysis (Bio-Rad), and the normalized relative mRNA expression was calculated against GAPDH expression. Sequences of gene-specific primers are listed in Supplementary Table S1.

### Immunohistochemistry

A total of 58 patient donor samples were used in the immunohistochemistry studies. The paraffin-embedded tissue blocks of coronary arteries were cut into 4 μm sections using a microtome, six serial sections (the first one of all five consecutive sections) were examined per lesion (for each antiserum) at ∼20 μm intervals, and a total of six images were analyzed per section for histological scoring. The sections were incubated for 1 h in 10% normal goat serum/phosphate buffered saline (PBS) solution and then incubated overnight with primary antibodies in 0.1% bovine serum albumin (BSA)/PBS solution in humid chambers at 4°C. The following primary antibodies and concentrations were used: mouse antihuman Wnt3a (Novus, at 1/100), mouse antihuman Wnt5a (Sigma Aldrich, at 1/100), rabbit antihuman Wnt5b (Abcam ab115563, 1/500), *α*-smooth muscle actin (*α*SMA) (Cedarlane, at 1/500), and cluster of differentiation 68 (CD68) (Sigma Aldrich, at 1:2,000). Secondary biotinylated goat antirabbit or antimouse IgG (Vector Labs, BA1000, BA9200 at 1:200) was applied, followed by the use of the Vectastain ABC Elite Kit (Vector Laboratories, Burlingame, CA), according to manufacturer protocols. Immunostaining was visualized by using a 1xDAB/H_2_O_2_ solution, subsequently counterstained with hematoxylin, and mounted with Permount (Sigma Aldrich). Immunostaining without primary antibodies or immunoabsorption with the respective antigens was performed by using negative controls. The serial sections were used for colocalization studies. Normal control studies were performed on normal sections of the coronary arteries.

Coronary plaques were semiquantitatively scored in a blinded manner for overall immunostaining based on the intensity and distribution throughout the plaque (Supplementary Table S2). The plaques were also assessed to determine the overall levels of calcification, fibrosis, lipids, remodeling, and inflammation (25). The relationship between Wnt3a, Wnt5a, and Wnt5b immunostaining and coronary plaque histopathological features was also assessed as previously described.

### Total protein extraction and Western blotting

Total proteins were extracted from tissues and cultured cells using a protein extraction buffer (8M Urea, 10 mM Tris, 1% SDS, pH 7.5). Briefly, frozen tissue was ground in liquid nitrogen, transferred into the extraction buffer, and then homogenized using a handheld homogenizer on ice. The cultured cells were washed with 1x PBS, scraped into a collection tube, and homogenized by passing them through a 22G needle 10 times. The extracted samples were spanned for 20 min at 4°C, and supernatants were collected; protein concentration was assayed using Protein DC reagents (Bio-Rad).

The total protein samples were separated on 10% SDS-PAGE and transferred into a PVDF membrane. The blot was blocked with 5% non-fat dry milk (BIO-RAD, #170-6404) in 1xTBST and incubated with primary antibodies overnight at 4°C in a blocking buffer. The following primary antibodies and concentrations were used: mouse antihuman Wnt3a (Novus, at 1/1,000), mouse antihuman Wnt5a (Sigma Aldrich, at 1/1,000), and rabbit antihuman Wnt5b (Abcam ab115563, 1/1,000). Secondary HRP-conjugated antibodies [Goat antimouse/Rabbit/Rat IgG (H + L) cross-absorbed, ThermoFisher, cat# G21040/G21234/A18870] were applied accordingly with primary antibodies. The SuperSignal™ West Pico PLUS Chemiluminescent Substrate (ThermoFisher, cat#34580) was applied and chemiluminescence images were taken and analyzed using the Chemidoc MP System (Bio-Rad).

### Cell culture

The cell lines presented in this study were normal primary human aortic SMC lines (ThermoFisher Scientific) that were cultured in Medium-231 including the Smooth Muscle Cell Differentiation Supplement (ThermoFisher Scientific) in the presence of 200 ng/mL of Wnt3a, Wnt5a, and Wnt5b (R&D Systems). Cells from passages 3 to 5 were used throughout the study.

### Migration assay

Cell migration was assessed by using 24-well Boyden chamber plates with an 8 µm pore size insert. Serum-free media were added to the bottom chamber and cells were seeded and treated with recombinant Wnt ligands in the top chamber for 24 h. The cells were washed with PBS, fixed with 4% paraformaldehyde, and stained with 0.05% crystal violet. Inserts were air-dried and the number of migrated cells was counted under a microscope.

### Measurement of ROS

CellROX™ Green Reagent (ThermoFisher Scientific) was used to measure mitochondrial superoxide, and the Amplex™ Red Hydrogen Peroxide/Peroxidase Assay Kit (ThermoFisher Scientific) was used to measure extracellular hydrogen peroxide, in accordance with the manufacturer's instructions. The cells were seeded in a 96-well black plate and treated with Wnt ligands for 2 h. Fluorescence intensity was measured using a TECAN Infinite® M200 Microplate reader (Tecan Group Ltd.) and normalized to total protein concentration.

### Calcification assay

Alizarin Red S staining was used to visualize the calcium deposition and calcification nodule formation. Briefly, SMCs were treated with or without 200 ng/mL Wnt proteins in an osteogenic medium containing 1 mM phosphate (OSM) for 10 days with fresh medium changed every 2–3 days. On the day of experimentation, the cells were fixed using 4% buffered formaldehyde and incubated with an Alizarin Red S stain solution (40 mM, pH4.2) for 20 min at room temperature. Calcification nodules were observed under microscopy, and phase-contrast images were taken with inverted microscopy. Calcium-bound Alizarin Red S was extracted by using a 10% cetylpyridinium chloride (Sigma) solution at room temperature for 60 min. Extracts were spanned for 5 min at 12,000 g and the supernatant was used for OD405mm reading on a spectrophotometer (BioTek). The cells were further washed with ddH_2_O and scraped into a 1.5-mL centrifuge tube in a cell lysis buffer (20 mM Tris.HCl pH7.4 with 2% SDS, 0.2M glycine) and heated at 80°C for 60 min occasionally with a vortex. The total protein content was assayed as described above and used for Alizarin Red S normalization. For comparison, we also measured calcium deposition using the Arsenazo III protocol following acid calcium extraction and observed data similar to those of Alizarin Red S quantification.

### Total cholesterol and cholesterol ester detection

SMCs were seeded in triplicates in 96-well culture plates and treated with 200 µg/mL of oxidized LDL (OxLDL) with or without 200 ng/mL Wnt proteins for 7 days in Medium-231 with fresh medium changes every 2–3 days. The OxLDL-treated cells for 7 days were used as controls. Total lipid was extracted using a 300 µL hexane: isopropanol (3:2 v/v) solution by incubating for 30 min on ice and was air-dried at 50°C. Total and free cholesterol were assayed using the Amplex Red Cholesterol Assay Kit (ThermoFisher Scientific). The lipid extract was reconstituted in a 120 µL 1xreaction buffer, 50 µL each was used for total and free cholesterol assay, and the cholesterol ester amount was calculated by subtracting free cholesterol from total cholesterol. The cells were lysed with 0.2N NaOH and protein concentration was assayed using the DC protein assay reagent (Bio-Rad) and used for normalization. A cholesterol standard curve was created by diluting a 2 mg/mL (5.17 mM) cholesterol reference standard into a 1x Reaction Buffer solution to produce cholesterol concentrations of 0–8 μg/mL (0 to ∼20 μM).

### Cholesterol efflux assay

SMCs were seeded in triplicates in 96-well culture plates and treated with 200 µg/mL of OxLDL with or without 200 ng/mL Wnt proteins for 48 h in Medium-231. The OxLDL-treated cells for 48 h were used as controls. On the day of experimentation, the treated cells were loaded with 25 μM TopFluor Cholesterol (Avanti Polar Lipids, Alabaster, Ala) overnight. Cholesterol efflux was assayed using 10 μg/mL APOA1 after 6 h. Cholesterol efflux was calculated by dividing the measured cholesterol in the media by the measured cholesterol in the cell and media, multiplied by 100%.

### Statistical analysis

An unpaired *t*-test was used to assess statistically significant differences in Wnt mRNA expression between diseased and normal arteries. A multivariate analysis was used to test the association of Wnt3a, Wnt5a, and Wnt5b immunohistochemical staining with the histological (overall levels of calcification, fibrosis, remodeling, inflammation, lipid score) and clinical parameters. Student’s *t*-test or one-way ANOVA, followed by Dunnett's multiple comparisons test, was used to assess differences between groups in the semiquantitative immunostaining experiments and in all *in vitro* assays. All values are presented as mean ± SEM. Statistical analyses were done using R (The R Project for Statistical Computing, Vienna, Austria), and all graphs were generated using GraphPad Prism 8 software. A score of *P* < 0.05 was considered statistically significant for all analyses.

## Results

### Expression of Wnt mRNAs and proteins in coronary plaques

**Figure 1:**
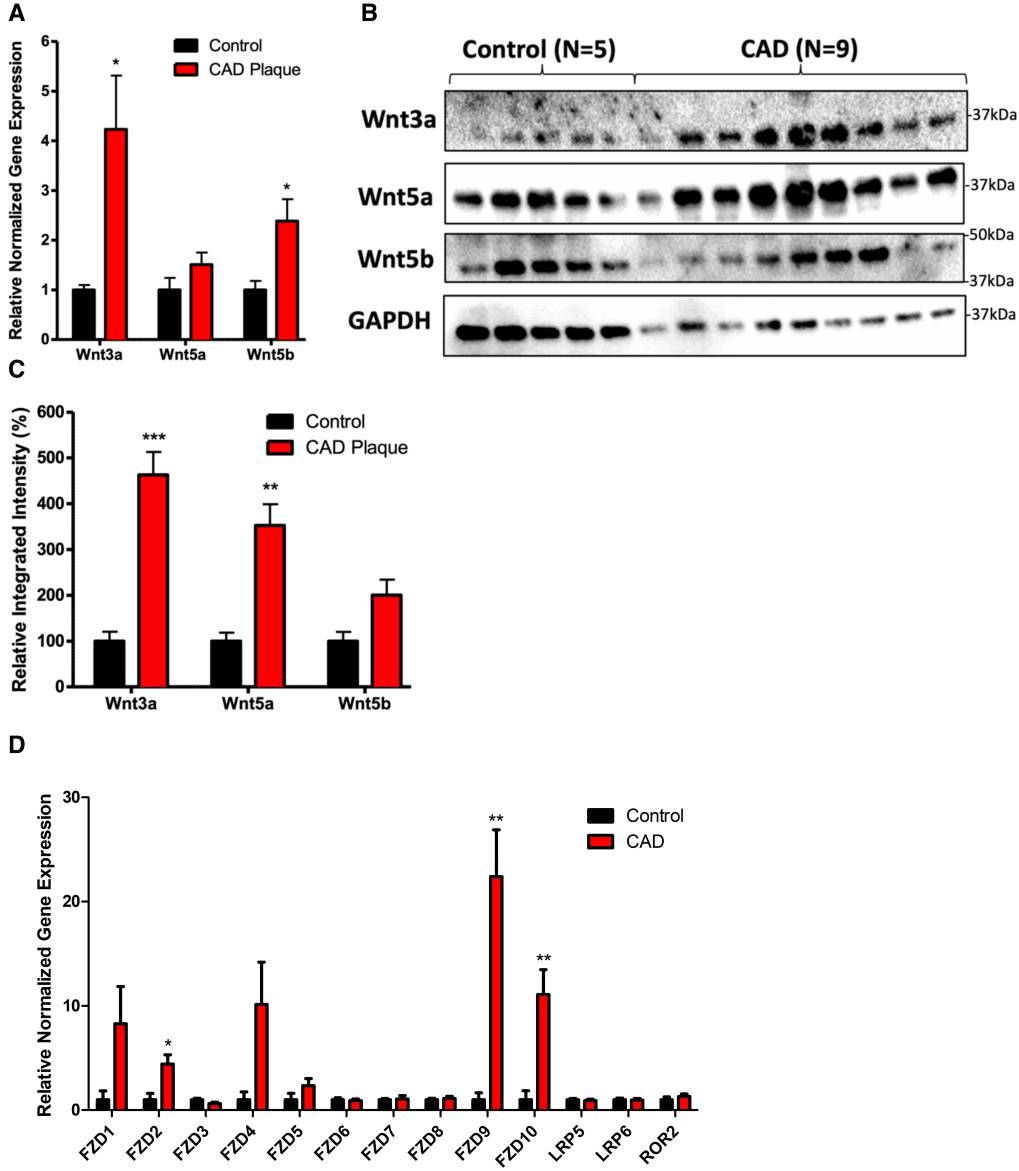
Coronary plaques have greater mRNA and protein expression of Wnt signaling proteins compared with healthy tissues. (**A**) An increased mRNA expression of Wnt3a and Wnt5b in diseased coronary arteries. The data are represented as mean ± SEM. *P < 0.05 compared with healthy tissues; *n* = 11 for coronary plaques and *n* = 7 for healthy tissues. Student’s *t*-test was used for statistical analysis. (**B**) A representative Western blot of Wnt proteins in diseased and healthy coronary arteries. (**C**) A densitometry analysis of the Western blot of Wnt protein expression in diseased and healthy coronary arteries. The data are represented as mean ± SEM. *P < 0.05, **P < 0.01, and ***P < 0.001 compared with normal tissues; *n* = 9 for coronary plaques and *n* = 5 for normal tissues. Student’s *t*-test was used for statistical analysis. (**D**) An increased mRNA expression of FZD2, FZD9, and FZD10 in diseased coronary arteries. The data are represented as mean ± SEM. *P < 0.05 compared with healthy tissues; *n* = 11 for coronary plaques and *n* = 7 for healthy tissues. Student’s *t*-test was used for statistical analysis. CAD, coronary artery disease.

There was a significant increase in the mRNA expression of Wnt3a and Wnt5b in coronary plaques compared with normal tissues (Figure 1A, *P* < 0.05). There was a significant increase in the protein expression of Wnt3a and Wnt5a in coronary plaques compared with normal control tissues (Figures 1B, C, *P* < 0.05). Although we observed an increase in Wnt5b protein expression, it did not reach statistical significance (*P* = 0.06). An analysis of Wnt receptor and coreceptor mRNA expression revealed an increased expression of FZD2, FZD9, and FZD10 in coronary plaques compared with normal tissues.

### Immunohistochemical analysis of Wnt3a

**Figure 2:**
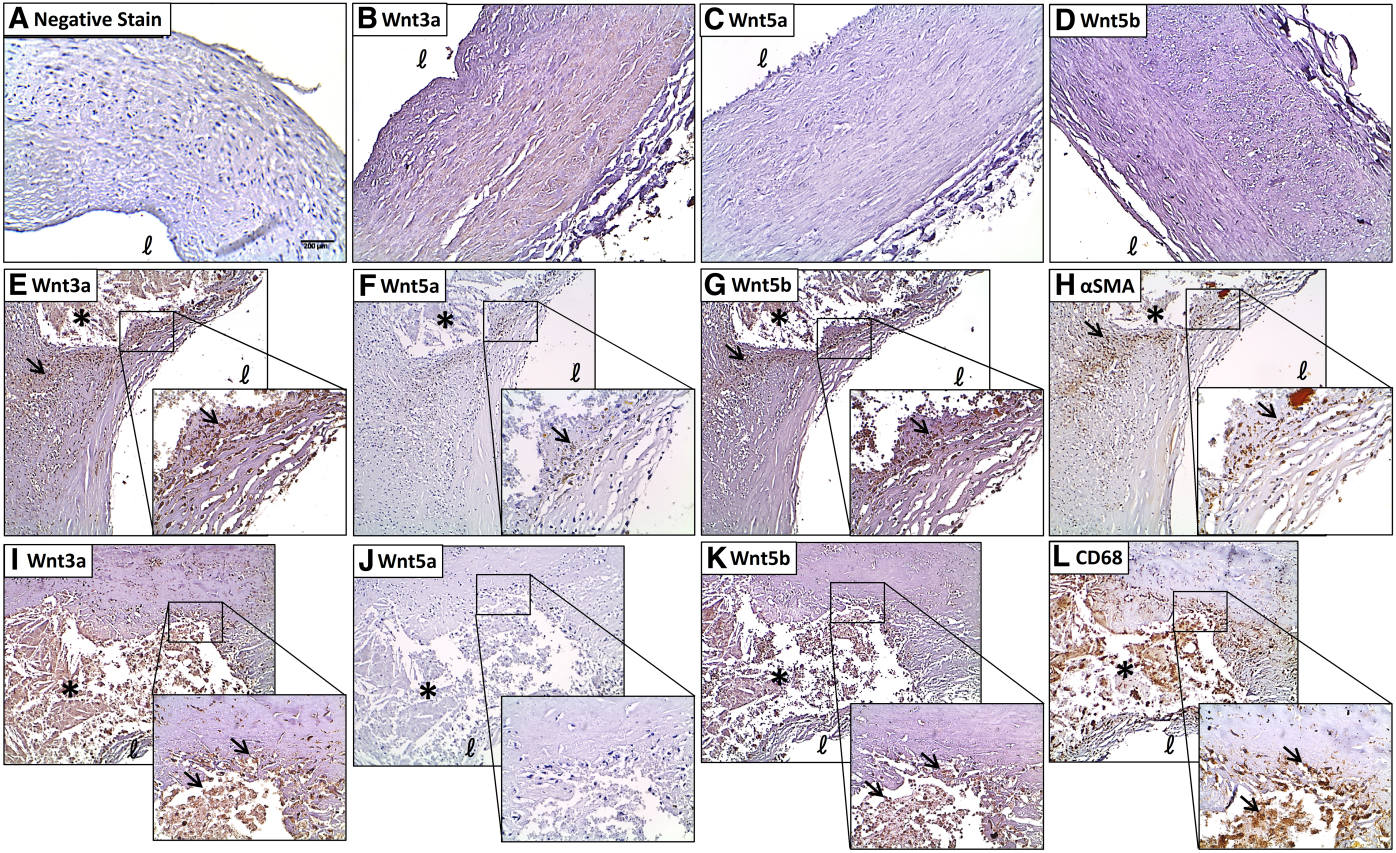
Coronary plaques have greater immunostaining of Wnt proteins compared with histologically normal tissues. (**A**) A negative stain control of the coronary artery. Very little Wnt3a (**B**), Wnt5a (**C**), and Wnt5b (**D**) immunostaining is seen in histologically normal coronary arteries. A strong Wnt3a staining is seen in the shoulder of the lesion around the lipid core and in the fibrous cap (**E**), and colocalized with αSMA (**H**) Wnt3a is colocalized with CD68 immunostaining around the lesion (**I**,**L**, respectively). Some Wnt5a immunostaining around the lipid core and in the fibrous cap (**F**) colocalized with αSMA (**H**) no significant colocalization of Wnt5a and CD68 immunostaining (**J**,**L**, respectively). A strong Wnt5b staining is seen in and around the lesion and in the fibrous cap (**G**) and colocalized with αSMA (**H**) colocalization of Wnt5b and CD68 immunostaining around the lesion (**K**,**L**, respectively). Scale bar = 200 μm. *Lipid core, **ℓ **= lumen. The arrows refer to significant immunostaining in and around the lesion.

Negative control sections immunostained with non-immune serum or reabsorbed with the respective antigens showed no immunoreactivity (Figure 2A). Faint immunoreactivity for Wnt3a was seen in the medial and intimal layers of normal coronary artery tissues with no evidence of plaque development (Figure 2B; 1.59 ± 0.19). Diseased coronary arteries widely expressed intense Wnt3a immunoreactivity, especially around calcified lesions (3.34 ± 0.31, *P* < 0.0001) and in the fibrous cap (3.13 ± 0.27, *P* = 0.0002) (Figure 2E). Primarily, Wnt3a was evident in myointimal cells (Figures 2E, H) and infiltrating macrophages (Figures 2I, L) as demonstrated by colocalization studies with a-SMC actin and CD68. A semiquantitative analysis of Wnt3a immunostaining revealed significantly elevated immunostaining in the diseased sections of coronary arteries compared with histologically healthy sections of the same artery (Supplementary Table S3, *P* < 0.0001). In tissues with severe remodeling, Wnt3a showed a moderately elevated expression (2.90 ± 0.23, *P* = 0.0022). Wnt3a was also abundantly expressed in infiltrating macrophages around the lipid core (3.28 ± 0.27, *P* < 0.0001). There was also an apparent presence of Wnt3a expression in the endothelial cells of microvessels (2.59 ± 0.26, *P* = 0.0346).

### Immunohistochemical analysis of Wnt5a

Weak immunoreactivity for Wnt5a was seen in the medial and intimal layers of normal coronary artery tissues with no evidence of plaque development (Figure 2C). Focal intense Wnt5a immunostaining was seen in coronary plaques, mainly around the lesion (Figure 2F) and in myointimal cells (Figures 2F, H). No significant Wnt5a staining was found in infiltrating foam cells (Figures 2J, L).

### Immunohistochemical analysis of Wnt5b

Weak immunoreactivity for Wnt5b was seen in the medial and intimal layers of normal coronary artery tissues with no evidence of plaque development (Figure 2D; 1.56 ± 0.17). A stronger Wnt5b immunostaining was seen around the lesion and in the fibrous cap (Figure 2G). Intense Wnt5b staining was found in myointimal cells (Figures 2G, H) and infiltrating macrophages (Figures 2K, L) around the lipid core. Semiquantitative analyses of the immunohistochemical data revealed significantly greater immunostaining for Wnt5b in diseased coronary arteries compared with normal coronary arteries (Supplementary Table S3, *P* = 0.0022). For example, compared with histologically normal coronary arteries, Wnt5b immunostaining was elevated in the areas of fibrosis (2.70 ± 0.29, *P* = 0.0055), remodeling (2.60 ± 0.30, *P* = 0.0143), and infiltrating macrophages (2.90 ± 0.22, *P* = 0.0007).

### Clinical associations with histological scoring

There was a significantly higher scoring for Wnt3a in coronary plaques from male patients compared with female patients (Supplementary Table S4, *P* = 0.0015). No significant differences were found for Wnt5a or Wnt5b histological scoring and other clinical parameters.

### Wnt proteins induced vSMC differentiation, migration, calcification, and oxidative stress

**Figure 3:**
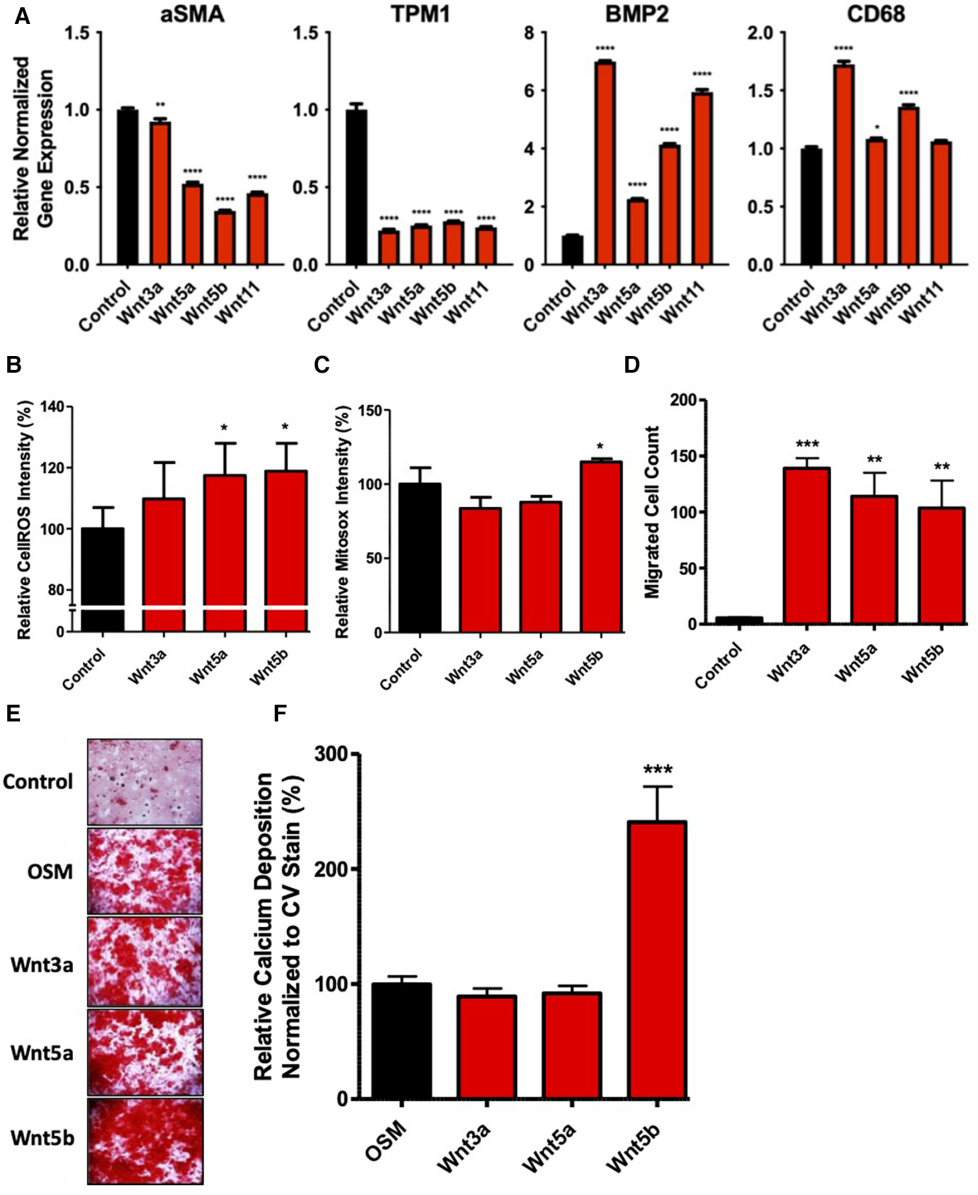
Wnt proteins induced an osteogenic differentiation of vascular smooth muscle cells and increased oxidative stress, cell migration, and calcification. (**A**) an mRNA expression of vSMC, osteogenic, and macrophage markers after treatment with 200 ng/mL Wnt proteins in vSMCs for 7 days. The data are represented as mean ± SEM. *P < 0.05, **P < 0.01, and ****P < 0.0001 compared with controls; *n* = 3 for all experimental groups. One-way ANOVA, followed by Dunnett's *post hoc* multiple comparisons test, was used for statistical analysis. Relative fluorescence intensity for the detection of (**B**) cellular reactive oxygen species and (**C**) mitochondria superoxides after treatment with 200 ng/mL Wnt proteins for 2 h. The data are represented as mean ± SEM. *P < 0.05 and **P < 0.01 compared with controls; *n* = 3 for all experimental groups. One-way ANOVA, followed by Dunnett's *post hoc* multiple comparisons test, was used for statistical analysis. (**D**) The migrated cell count of SMCs after treatment with 200 ng/mL Wnt proteins in serum-free media for 24 h. The data are represented as mean ± SEM. *P < 0.05, **P < 0.01, and ***P < 0.001 compared with controls; *n* = 3 for all experimental groups. One-way ANOVA, followed by Dunnett's *post hoc* multiple comparisons test, was used for statistical analysis. Representative images (**E**) and quantification of a calcification stain (**F**) of vSMCs after treatment with 200 ng/mL Wnt proteins in OSM for 7 days. The data are represented as mean ± SEM. **P < 0.01 and ***P < 0.001 compared with controls; *n* = 3 for all experimental groups. One-way ANOVA, followed by Dunnett's *post hoc* multiple comparisons test, was used for statistical analysis. OSM, osteogenic media; SFM, serum-free media; vSMC, vascular smooth muscle cells.

SMCs treated with Wnt recombinant proteins reduced the expression of SMC markers alpha-smooth muscle cell actin and tropomyosin 1 (TPM1) (Figure 3A). Furthermore, all Wnt proteins were found to increase the expression of the osteoblast marker bone morphogenetic protein 2 (BMP2) (*P* < 0.0001), with the greatest fold change seen for Wnt3a. The latter was also found to increase the expression of macrophage marker CD68 (*P* < 0.0001), along with Wnt5a (*P* < 0.05) and Wnt5b (*P* < 0.0001). Interestingly, only the non-canonical ligands Wnt5a and Wnt5b induced significant oxidative stress in vSMCs (*P* < 0.05, Figure 3B), with only Wnt5b showing a significant generation of mitochondrial superoxides (*P* < 0.05, Figure 3C). Treatment of vSMCs with Wnt3a, Wnt5a, and Wnt5b resulted in a profound increase in migratory ability (Figure 3D), with Wnt3a having the largest effect (*P* < 0.01). SMCs treated with Wnt5b showed significant calcium deposition (*P* < 0.05), with no changes seen in cells treated with Wnt3a or Wnt5a (Figures 3E, F).

### Wnt proteins impair cholesterol handling in vSMCs

**Figure 4:**
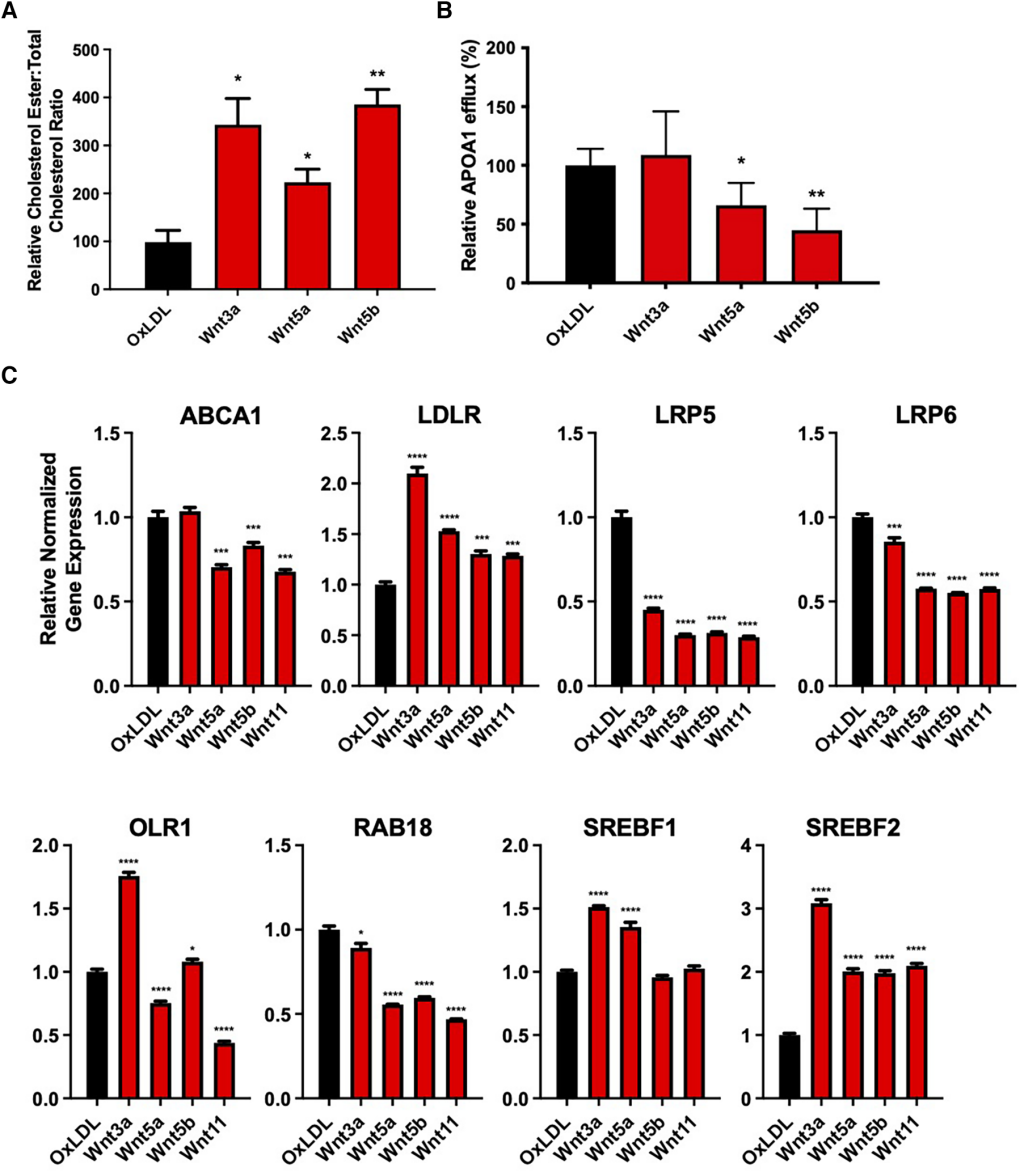
Wnt proteins impaired cholesterol handling in vascular smooth muscle cells. (**A**) Cholesterol ester to total cholesterol ratio in vSMCs treated with 200 µg/mL oxLDL and/or 200 ng/mL Wnt proteins for 7 days. The data are represented as mean ± SEM. *P < 0.01 and **P < 0.01 compared with controls; *n* = 3 for all experimental groups. Student's *t*-test was used for statistical analysis. (**B**) ApoA1-mediated cholesterol efflux in vSMCs treated with 200 ug/mL oxLDL and/or 200 ng/mL Wnt proteins for 48 h. The data are represented as mean ± SEM. *P < 0.01 and **P < 0.01 compared with controls; *n* = 3 for all experimental groups. Student's *t*-test was used for statistical analysis. (**C**) an mRNA expression of cholesterol handling genes after treatment with 200 µg/mL oxLDL and/or 200 ng/mL Wnt proteins for 7 days. The data are represented as mean ± SEM. *P < 0.01, **P < 0.01, ***P < 0.001, and ****P < 0.0001 compared with controls; *n* = 3 for all experimental groups. One-way ANOVA, followed by Dunnett's *post hoc* multiple comparisons test, was used for statistical analysis. vSMC, vascular smooth muscle cells.

SMCs treated with OxLDL and Wnt3a, Wnt5a, or Wnt5b showed an increased cholesterol ester to total cholesterol ratio, compared with cells treated with OxLDL alone (*P* < 0.05, *P* < 0.05, and *P* < 0.01, respectively, Figure 4A). Furthermore, OxLDL-treated vSMCs, in conjunction with non-canonical Wnt5a or Wnt5b, significantly reduced apolipoprotein A1 (APOA1)-dependent cholesterol efflux, with no changes seen for Wnt3a (*P* < 0.05, Figure 4B).

Non-canonical Wnt5a and Wnt5b reduced ATP binding cassette subfamily A member 1 (ABCA1) mRNA expression, with no changes seen for canonical Wnt3a (*P* < 0.0001, *P* < 0.001, respectively, Figure 4C). All proteins were found to significantly reduce the expression of plasma membrane receptor proteins, low-density lipoprotein receptor–related protein 5 (LRP5) (*P* < 0.001) and LRP6 (*P* < 0.001). There was also an increased expression of low-density lipoprotein receptor (LDLR) mRNA after treatment with all Wnt proteins (*P* < 0.001), while only Wnt3a and Wnt5b increased the expression of oxidized low-density lipoprotein receptor 1 (OLR1) mRNA. In addition, all Wnt proteins significantly reduced the expression of the cholesterol transport ras-related protein rab-18 (RAB18), which is involved in lipid transport. Lastly, Wnt3a (*P* < 0.0001) and Wnt5a (*P* < 0.0001) increased the expression of the cholesterol synthesis gene, sterol regulatory element-binding transcription factor 1 (SREBF1), while all Wnt proteins increased the expression of SREBF2 (*P* < 0.0001). A summary of these findings is shown in Supplementary Figure S1.

## Discussion

Previous studies suggested a prominent role for Wnt5a and the Wnt coreceptors LRP5/6 in the pathogenesis of CAD. However, the role of other Wnts and the mechanisms by which Wnts affect CAD remain poorly understood (7, 13, 14). Wnt3a belongs to the family of canonical Wnt ligands, which promote the translocation of β-catenin to the nucleus. Wnt5a and Wnt5b belong to the family of non-canonical Wnt proteins, which can activate several pathways, including the calcium and cell polarity pathways. Since these Wnt proteins activate different intracellular mechanisms, they may have differential effects on cell phenotype and function. Here, we show that Wnt3a, Wnt5a, and Wnt5b proteins are abundantly expressed in human coronary plaques compared with healthy controls, while only Wnt3a and Wnt5b mRNA are significantly upregulated in diseased tissues. In addition, we also show that these ligands are involved in the pathogenesis of CAD by altering vSMC phenotype, migration, calcification, and cholesterol handling.

Wnt3a is present in vSMCs and the macrophages of human coronary plaques, and the stimulation of Wnt3a in mouse SMCs increases β-catenin nuclear localization and TCF activity (26). Wnt3a has also been shown to induce a synthetic phenotype in SMCs and increase their migration (27). Wnt5a has previously been shown to be expressed in carotid atherosclerotic lesions compared with healthy tissues in both mice and humans (15–17). However, the composition of carotid and coronary plaques has been suggested to differ in several aspects such as plaque erosion, calcification, fibrous cap thickness, and most importantly, macrophage accumulation (28). Moreover, none of the abovementioned studies comprehensively determined the role of these important molecules in vSMC calcification and lipid handling. In this study, we not only detail the expression of these three important molecules and their receptors in CAD but also provide strong evidence for their involvement in vSMC function and fate. This is also the first time that Wnt5a has been shown to be elevated in patients with CAD and expressed largely in myointimal cells. This is also the first report of Wnt5b expression in macrophages and myointimal cells around the atherosclerotic lesion.

During atherosclerotic plaque development, endothelial dysfunction results in a phenotypic switch of SMCs from a contractile quiescent state to an active synthetic state, resulting in their expansion and migration to the intimal layer (29). This is, in part, mediated by the generation of ROS, which signals SMC proliferation, migration, and hypertrophy (30). In this study, we show that all Wnt ligands induced a migration of SMCs, while only the non-canonical Wnt ligands caused significant ROS generation. Our findings are in agreement with those of previous reports, which showed that an activation of Wnt signaling resulted in significant ROS generation, in part, through the non-canonical Wnt/planar cell polarity signaling pathway (31). Others suggest that increased intracellular calcium release generates ROS, which mediates Wnt/β-catenin signaling (32). Our results indicate that Wnt ligands promote several aspects of atherogenesis in vSMCs, including increased cell migration, calcium deposition, and ROS generation, which may be a result of differentiation into osteoblast-like or macrophage-like cells.

The differential effects on gene expression observed in response to Wnt stimulation can be attributed to the multifaceted nature of Wnt signaling pathways and their context-dependent activation. Shared effects, such as increased CD68 expression, may result from converging downstream signaling events triggered by both canonical and non-canonical pathways, reflecting common processes like inflammation that are fundamental to atherosclerotic disease (33, 34) It has been reported that the convergence can occur at level B-catenin, and downstream gene activation depends on the B-catenin cofactor involved. In addition, some Wnt ligands have regulatory effects on other Wnt pathways. For example, in cancer, Wnt3a has been shown to activate both Wnt/Ca++ and Wnt/β-catenin (35). On the other hand, many reports indicate that Wnt5a can either activate or repress B-catenin activation depending on the context (35, 36)*.* Wnt signaling outcomes vary because of pathway interactions and cellular context, with shared effects like inflammation stemming from both canonical and non-canonical pathways, and distinct effects being B-catenin cofactor and ligand-specific. While all the Wnt ligands used in this study significantly increased the mRNA expression of BMP2, the differential fold change is difficult to interpret. Wnt3a has been shown to induce mineralization in chondrocytes (37). This study suggests that other cofactors such as VEGF may be the reason for the significant mineralization effects, in keeping with the fact that specific contexts may differentially activate particular downstream genes from Wnt ligands.

Impairments in cholesterol handling are a hallmark of atherosclerosis (38–40). In response to injury, medial vSMCs are recruited from the media and undergo pathogenic differentiation to either mesenchymal-like cells, macrophage-like cells, foam-like cells, or osteoblast-like cells and produce an extracellular matrix to help stabilize plaques (41). Culturing vSMCs in the presence of cholesterol particles has been previously shown to promote differentiation into macrophage-like or foam-like cells through changes in inflammatory gene expression and phagocytosis mechanisms (42). In fact, a previous study demonstrated that approximately 50% of foam cells in lesions were derived from vSMCs (43), suggesting that vSMCs play a substantial role in plaque development. Our results corroborate these findings, showing that both canonical and non-canonical Wnt ligands induce the expression of macrophage and osteogenic markers. We also show that Wnts increase cellular cholesterol ester content, while reducing APOA1-dependent cholesterol efflux, suggesting that these cells are more prone to become foam-like cells via impaired cholesterol handling. Interestingly, Wnt3a has been shown to rescue the proatherogenic phenotype in LRP6 mutant mice by reducing the levels of plasma triglyceride, total cholesterol, and LDL-C (44), highlighting the importance of the LRP6 signaling axis in maintaining normal cholesterol levels.

Cholesterol handling by vSMCs is largely affected by several groups of plasma membrane proteins. Two major cell surface receptors affect cholesterol influx, and these are LDLR and OLR1, which import LDL and OxLDL particles, respectively. An increased expression of these receptors removes excess cholesterol from the plasma; however, this may result in elevated cellular cholesterol levels and can have detrimental effects on cell phenotype and function (45, 46). In this study, it was found that only Wnt3a and Wnt5b increased the mRNA expression of OLR1, while all proteins increased the expression of LDLR in vSMCs. There was also a reduction in ABCA1 mRNA expression, which plays an important role in reverse cholesterol transport. The changes in transcriptional activity of these genes may account for the impairment in APOA1-dependent cholesterol efflux found after treatment with the non-canonical Wnt proteins (47). Lastly, it was found that all Wnt proteins used in this study reduced the expression of the Wnt receptors LRP5 and LRP6 (48, 49). Deficiency of LRP5 in hypercholesterolemic mice was found to increase lipid infiltration, plasma inflammatory cytokine production, and total cholesterol levels (48). A downstream analysis revealed that LRP deficiency was associated with a downregulation of β-catenin immunostaining and LDLR mRNA expression. These findings are corroborated by other groups, which show that differentiating vSMCs in atherosclerosis lesions have a reduced expression of SMC markers and ABCA1 cholesterol receptor gene expression (43). In addition, the Wnts used in this study also affected the cholesterol synthesis genes SREBF1 and SREBF2. When cholesterol levels are depleted, sterol regulatory element-binding proteins (SREBs) are recruited to the Golgi apparatus, where they are cleaved and translocated to the nucleus to activate genes involved in cholesterol, fatty acid, and triglyceride synthesis (50). Here, we showed that Wnt proteins upregulated SREBP1 and SREBP2 mRNA to increase free fatty acid and cellular cholesterol levels, respectively. Our results suggest that Wnt proteins enhance cholesterol accumulation in the cell by increasing uptake and reducing reverse cholesterol transport.

The Wnt signaling pathway plays a complex role in both cardiovascular development and disease progression. Here, we clearly describe the expression of Wnt3a, Wnt5a, and Wnt5b and their receptors in human coronary plaques. Furthermore, our *in vitro* studies show a pathogenic role of Wnt3a and Wnt5b in human vSMC migration and ROS generation. More studies are encouraged to target and inhibit these Wnt ligands in vSMCs and animal models to investigate their therapeutic potential.

## Data Availability

The original contributions presented in the study are included in the article/Supplementary Materials, and further inquiries can be directed to the corresponding author.
