# Supplementary material for: Significance of the Wnt signaling pathway in coronary artery atherosclerosis
Source: Front Cardiovasc Med. 2024 Mar 22;11:1360380. doi: 10.3389/fcvm.2024.1360380 (PMC10995361; doi:10.3389/fcvm.2024.1360380)
Supplement: Supplementary file 1 [file Datasheet1.docx]

**Supplemental Materials**

**Supplemental Table I. Sequences of primers used for RT-PCR analysis.**

| **Gene** | **Forward Sequence** | **Reverse Sequence** |
| --- | --- | --- |
| WNT3A | ACTCGGATACTTCTTACTCCTCTGC | GAGCCCAGGGAGGAATACTG |
| WNT5A | CTTCCAAGTTCTTCCTAGTGGCTTT | TGAACAGGGTTATTCATACCTAGCG |
| WNT5B | GGAAACTGTCAGTCCCAGGGC | CTGCACCGGGTTCAAAGCTAAT |
| αSMA | CTATGCCTCTGGACGCACAACT | CAGATCCAGACGCATGATGGCA |
| TPM1 | TTGAAAGCATTAATGGCTGCAGAGG | TAGTTACTGACCTCTCCGCAAACTC |
| BMP2 | TCCTTTGACCAGAGTTTTTCCATGT | GAAGCAGCAACGCTAGAAGACA |
| CD68 | CTGTACTGAACCCCAACAAAACCAA | TGTAGCTCAGGTAGACAACCTTCTG |
| LDLR | TATCAGAAGACCACAGAGGATGAGG | CGTCATCCTCCAGACTGACCATC |
| OLR1 | ACCTAAAGATCCAGACTGTGAAGGA | TAATGGTCACTACTAATCCCAGGCA |
| LRP5 | GACCCACTGGACAAGTTCATCTAC | GCTCAGAGAGGTCAAAACAAAGG |
| LRP6 | TATTCTTCAAACAGTCCTTCCACTC | GAGCATAGTCACTGTCACAAACATC |
| ABCA1 | TATGAGGACAACAACTACAAAGCCC | AGATAATGCGGGAAAGAGGACTAGA |
| SREBP1 | CGGAGCCATGGATTGCACTTTC | CCCAGCATAGGGTGGGTCAAATAG |
| SREBP2 | GAGACCATGGAGACCCTCACG | TCAGGGAACTCTCCCACTTGATTAC |
| RAB18 | ACGTGCTAACCACCCTGAAGAT | TCAAACGTATCATCTGTGAACCTCAA |
| GAPDH | TGCACCACCAACTGCTTAGC | GGCATGGACTGTGGTCATGAG |

**Supplemental Table II. Reference chart used for semi-quantitative analysis of immunohistochemistry experiments based on intensity and distribution.**

| **Score** | **Distribution** | **Intensity** |
| --- | --- | --- |
| **0** | No stain | No stain |
| **1** | <5% of ECM or cells | Faint |
| **2** | <25% of ECM or cells | Light brown |
| **3** | <50 of ECM or cells | Medium brown |
| **4** | <75% of ECM or cells | Deep brown |
| **5** | >75% of ECM or cells | Dark brown |

**Supplemental Table III. Analysis of Wnt3a and Wnt5b immunohistochemical scoring in normal and diseased human coronary plaques.**

|  | **Wnt3a** | | **Wnt5b** | |
| --- | --- | --- | --- | --- |
| **Parameter** | **Mean ± SEM** | **P-value** | **Mean ± SEM** | **P-value** |
| **Normal segment of diseased**  **artery** | 1.59 ± 0.19 |  | 1.56 ± 0.17 |  |
| **Calcification** | 3.34 ± 0.31 | < 0.0001 | 2.43 ± 0.26 | NS |
| **Fibrosis** | 3.13 ± 0.27 | 0.0002 | 2.70 ± 0.29 | 0.0055 |
| **Lipid** | 3.10 ± 0.26 | 0.0003 | 2.17 ± 0.23 | NS |
| **Remodeling** | 2.90 ± 0.23 | 0.0022 | 2.60 ± 0.30 | 0.0143 |
| **Inflammation** | 3.28 ± 0.27 | < 0.0001 | 2.90 ± 0.22 | 0.0007 |
| **Microvessel** | 2.59 ± 0.26 | 0.0346 | 2.45 ± 0.21 | NS |

**Supplemental Table IV. Analysis of Wnt3a and Wnt5b immunohistochemical scoring and correlation between clinical variables in human coronary plaques.**

|  | **Wnt3a** | | **Wnt5b** | |
| --- | --- | --- | --- | --- |
| **Parameter** | **Spearman R** | **P-value** | **Spearman R** | **P-value** |
| **Sex* (Female vs. Male)** | 2.2 ± 0.33 vs.  3.7 ± 0.25 | 0.0015 | 2.85 ± 0.35 vs.  2.89 ± 0.27 | NS |
| **Age (yrs)** | -0.06 | NS | -0.11 | NS |
| **Weight (kg)** | -0.02 | NS | -0.29 | NS |
| **Total cholesterol** | -0.26 | NS | 0.51 | NS |
| **LDL** | -0.33 | NS | 0.52 | NS |
| **HDL** | 0.11 | NS | 0.29 | NS |
| **Triglycerides** | 0.17 | NS | 0.14 | NS |
| **Hba1c** | -0.18 | NS | -0.05 | NS |

*Unpaired students t-test was used to evaluate difference between male and female sex and histological scoring. Data is presented as immunohistochemical score mean ± SEM.

Abbreviations: BMI: body mass index; HDL: high-density lipoprotein; LDL: low-density lipoprotein,

**Supplemental Figure 1. Schematic representation of the effects of Wnt signaling pathways on cholesterol handling in smooth muscle cells.**
